# How, when, and to what degree do people with alcohol dependence recover their psychological wellbeing and quality of life? The Madrid Recovery Project

**DOI:** 10.3389/fpsyt.2023.1130078

**Published:** 2023-06-15

**Authors:** Gabriel Rubio, Laura Esteban Rodríguez, Ana Sion, Lilian Ramis Vidal, María J. Blanco, Amelia Zamora-Bayon, Marta Caba-Moreno, Ana I. Macias-Molina, Dolores Pérez-Sánchez, Enrique Rubio-Escobar, Joaquín Ruiz-Diez, Marta Marin, Francisco Arias, David Lora, Rosa Jurado-Barba

**Affiliations:** ^1^Biomedical Research Institute, Hospital 12 de Octubre, Madrid, Spain; ^2^Medicine Faculty, Complutense University of Madrid, Madrid, Spain; ^3^Primary Care Research Network on Addictions (RIAPAd), Madrid, Spain; ^4^Psychology Faculty, Complutense University of Madrid, Madrid, Spain; ^5^Alcoholics Federation of the Community of Madrid (FACOMA), Madrid, Spain; ^6^Faculty of Statistical Studies, Complutense University of Madrid, Madrid, Spain; ^7^Department of Psychology, Education and Health Science Faculty, Camilo José Cela University, Madrid, Spain

**Keywords:** alcohol use disorder, quality of life, psychological wellbeing, recovery stages, negative emotionality, coping strategy, Madrid Recovery Project

## Abstract

**Introduction:**

The consensus on recovery from alcohol use disorder (AUD) has shifted toward encompassing psychological wellbeing and quality of life dimensions. However, few studies have explored the long-term recovery process and its dimensions, timing, styles, and modes. The aim of this study was to investigate the extent, timing, and process of psychological wellbeing and quality of life recovery in alcohol use disorder (AUD) patients, as well as the relationship with classic dimensions of AUD recovery.

**Method:**

A cross-sectional study has been carried out with 348 participants with AUD, in different abstinence periods (1 month–28 years), and 171 control subjects. Participants underwent a psychological evaluation, which included self-informed measures of psychological wellbeing, quality of life, negative emotionality, and coping strategies related to alcohol consumption avoidance. Statistical analysis included linear and non-linear regression models between psychological dimensions and maintenance of abstinence, as well as matching the scores of the sample with AUD to those of controls. Scatter plots were used to explore inflection points. In addition, mean comparison tests were performed between participants with AUD and controls and by gender.

**Results:**

In general, according to the regression models, there were pronounced increases in indices of wellbeing and coping strategies (and pronounced decreases in negative emotionality) during the first 5 years of abstinence, followed by less pronounced improvements. The matching of AUD subjects in wellbeing and negative emotionality indices with controls occurs at different times: (a) 1 year or less: physical health; (b) 1–4 years: psychological health; (c) 4–10 years: social relationships, wellbeing, and negative emotionality; and (d) more than 10 years: autonomy and self-acceptance. There are statistically significant differences by gender for the negative emotionality and physical health variables.

**Conclusion:**

Recovery from AUD is a long process that involves improvements in wellbeing and quality of life. Four stages can be described in this process, with the most pronounced changes occurring during the first 5 years of abstinence. However, AUD patients take more time to obtain similar scores to controls in several psychological dimensions.

## 1. Introduction

The term “recovery” has been broadly studied and associated with alcohol and other substance use disorder processes (1). Nevertheless, at the present time, we still lack a solid consensus on the meaning of recovery and how it should be measured (1–5). For decades, a large part of treatment perspectives has focused on abstinence maintenance and attendance to self-help groups, such as 12-step programs, as measures of recovery. This vision might have led to a reductionist view of recovery. Nonetheless, the biopsychosocial models propose a more comprehensive view of recovery, which includes the management of different resources and abstinence maintenance strategies aimed to improve personal and familial assets, together with psychological and relational health improvements (6).

Among the newer and broader definitions of recovery, SAMHSA’s (7) one stands out, describing the recovery process as the change that allows individuals to improve their health and wellbeing, to drive their own life, and to boost their potential. A more current definition is offered by Witkiewitz et al. (5), which is based on previous definitions and a recent empirical study. The authors propose that recovery is a process of behavioral change characterized by improvements in biopsychosocial functioning and life purpose (5). People in recovery usually experience relevant increases in physical, emotional, and relational health. Moreover, evidence of these improvements is shown in studies from several countries, such as Canada (8, 9), the United States (10), the United Kingdom (11), and Australia (12, 13). Recovery aspects seem to be related to factors such as lifestyle changes, wellbeing, and available resources or assets (as understood within the recovery capital framework) (3, 6, 14–19).

The ecological framework of recovery capital (RC) considers the various interrelated factors that promote recovery. Thus, RC refers to the total sum of the own resources that one can use to initiate and maintain recovery from alcohol and other drug dependence (20). From Granfield and Cloud’s (20) proposal, resources are distinguished at the individual and societal levels. We have focused on individual resources. Hennessy’s (21) systematic review of RC sets out that individual resources include those as follows: (I) physical capital (tangible capital, e.g., material resources such as money or the availability of a public treatment center); (II) human capital (personal characteristics to achieve goals: e.g., knowledge, interpersonal skills, emotional stability, or mental health); (III) growth capital it is based on a person’s innate desire to grow and develop in a positive direction. It refers to the external and internal resources that support this growth. The recovery process attempts to remove obstacles to further growth so that it initiates and continually supports further growth toward recovery progress [see Hennessy’s review (21)]. Additionally, recovery capital models include a dimension that refers to personal recovery. This dimension combines physical capital and human capital. The biaxial recovery model of Kelly and Hoeppner (3) proposes that recovery is constituted in two axes: (I) one related to the substance, which they call ‘remission’ and refers to withdrawal or abstinence time and (II) another that alludes to recovery capital from the framework of Cloud and Granfield (20). According to this biaxial recovery model (3), the relationship between remission and recovery capital must be reciprocal. Therefore, more time in remission will increase the positive consequences that flow from it. At the same time, increasing these positive consequences, i.e., possessing greater recovery capital, will increase the likelihood of long-term remission. Thus, it appears that recovery capital is a framework that is gaining momentum in the study of recovery and has gained interest in the treatment field and in addiction recovery research, by providing a broad perspective on the process (21). For example, the UK government’s addiction agenda has shifted from a focus on harm management and a primarily disease-based view of addiction, to a focus on building recovery capital and fostering the role of patient activation and self-management to enhance recovery (22).

Despite the increasing need to expand and detail the dimensions of recovery, most studies published until now show a series of methodological limitations that make it difficult to obtain broader a vision of the recovery process. There is abundant literature on the first step of recovery and on factors that predict the acquisition of abstinence, whereas quantitative studies on long-term recovery characteristics are quite scarce (6, 15, 17).

A small number of studies have addressed recovery considering long-term changes in psychological processes and a perspective that goes beyond the reduction of symptoms. Among them, we can outline the study of Kelly et al. (16), which includes participants from community samples, or the study of Witbrodt et al. (23) in clinical samples. Moreover, studies usually include a small sample size. A recent systematic review (1) showed that from the 36 studies reviewed, only 11 included samples superior to 100 participants.

In relation to studies aimed at recovery in terms of wellbeing and quality of life, the results from community samples by Kelly et al. (16) show an improvement in quality of life and psychological wellbeing, as the abstinence period increases, especially in the first 5 years. The review by Donovan et al. (24) reports that alcohol-dependent people experience improvements in their quality of life throughout treatment and with abstinence, both in the short and long term. This review notes that “despite these improvements, many individuals’ QoL is unlikely to equal or exceed that of normative groups” (24).

Regarding the temporal sequence of psychological dimensions, changes, and quality of life improvement in recovery, the evidence published to date indicates that most variables improve during the 1st month/year after ceasing alcohol consumption (25–27). For instance, Laudet et al. (25), in a cross-sectional study, observed that distress ameliorated rapidly during the 1st year and continued to improve at a slower pace until the 3rd year. Dennis et al. (26), in a prospective study, observed that quality of life variables improved over time; however, at the 3rd year, an exacerbation of the psychological distress was found. In addition, our research group found in a 6-year follow-up study of outpatients with alcohol dependence that negative emotionality variables diminished during the first 3 years and then stabilized, while meaning in life kept improving until the end of the follow-up period (27, 28).

These findings have led us to propose a sequence in psychological recovery that would initiate with lifestyle behavioral changes, such as developing avoidance strategies against alcohol use, followed by improvements in clinical dimensions such as anxiety, sadness, and impulsivity and an overlapped increase in meaning in life (28). Nonetheless, a more detailed characterization of AUD recovery and information on how individuals with AUD recover psychological wellbeing is required. Moreover, the extent of improvement in wellbeing, quality of life, depression, or anxiety during the recovery process is still to be determined, and the equation of these dimensions to normative groups is yet to be explored. As Kelly et al. (16) pointed out, regarding long-term recovery, the question is more about the process, how and to what extent people with AUD experience improvement in wellbeing, and it is less about whether it takes place or not.

Learning about the elements of recovery could serve to identify the milestones or the turning points that might indicate the periods of increased vulnerability, resilience, or personal growth during the adaptation period. The competence in this field should include the understanding of the nature, level, the changing periods of different indexes that reflect wellbeing and functioning of the individuals. At the same time, this can provide information on the services needed to maintain the long-term recovery in several junctures and periods and personalized attention for the patient.

To achieve a deeper knowledge on the recovery course and the different psychological dimensions and quality of life in patients with severe AUD, research in a metropolitan area of Madrid was carried out, at the Public Alcohol Dependence Treatment Program of 12 de Octubre Hospital. The aim of this study was to examine the complexity of the recovery process in a clinical sample by analyzing various dimensions. The study examined the involvement of abstinence time understood as the “remission” axis proposed by Kelly and Hoeppner (3) in the Recovery Capital axis (3) and other clinical variables (negative emotionality and impulsivity). This provides a clinical perspective on the different temporal moments of the recovery process in patients with AUD in abstinence.

Abstinence time was utilized as a follow-up measure for recovery, given its relevance in cognitive-behavioral treatment that patients attended and previous literature supporting its role as a factor of recovery (15). In contrast to other studies, this research measured clinical manifestation variables, including negative emotionality and symptoms such as anxiety, depression, experiential avoidance, and impulsivity, as well as quality of life and psychological wellbeing. Additionally, recovery capital and coping strategies in relation to alcohol use and dependence were assessed. Furthermore, the possibility to evaluate patients at different stages of the recovery process would allow to observe the evolution of changes in the studied variables, having a control group of healthy participants would also enable a better view of patients’ recovery.

## 2. Materials and methods

### 2.1. Experimental design

A cross-sectional study of a control and case study was carried out. On the one hand, 348 abstinent individuals with alcohol use disorder (AUD), that attended outpatient programs, either at the Psychiatry Service of 12 de Octubre Hospital, or self-help groups of the Community of Madrid, were included in the study. On the other hand, 171 healthy controls took part in the study, and they were matched in age, gender, and educational level.

All procedures were approved by the 12 de Octubre Ethics Committee.

### 2.2. Participants

With respect to participants with AUD, the sample consisted of 348 participants in a situation of complete abstinence (abstinence time range: 1 month–28 years). All participants were attending treatment aimed at abstinence, either at the public program of the Hospital 12 de Octubre or in mutual help groups. Participants with less than 2 years of abstinence attended the therapeutic program of the Psychiatry Service of the Hospital 12 de Octubre on an outpatient basis. Details regarding the therapeutic program can be read in Rubio et al. (27). This is a public treatment program (financed and managed by the public health system, so it is free of charge) with a duration of 2 years. This program sequentially addresses different aspects of abstinence-directed recovery: detoxification and motivation for abstinence; relapse prevention, social skills, consolidation of healthy habits and lifestyle, and preparation for discharge. Subsequently, it can be continued by participation in mutual help groups. Participants proceeding from 12 de Octubre Hospital were recruited in the treatment context. Senior adjunct psychiatrists, head of the program, asked them to join the study. They emphasized that participation was voluntary, and, under no circumstances, it would affect their treatment. Those who accepted to carry out the study were individually evaluated in the hospital’s facilities.

To complete the sample of patients with more than 2 years of abstinence, those attending mutual help groups were invited to participate. Specifically, three associations of the Federation of Alcoholics of the Community of Madrid (FACOMA) and three of Alcoholics Anonymous (AA) participated in the study. In this case, the recruitment was done by the psychologist in charge of the therapeutic groups and the psychological assistance in FACOMA’s locations. Recruitment conditions were similar to the previous one: They had explicit indications regarding voluntary participation, without treatment repercussions. Individuals were assessed at each location of the FACOMA. For individuals coming from AA groups, recruiting was carried out through a representative that was responsible for the invitation to the study. Once they manifested interest in the study, a senior psychiatrist from 12 de Octubre Hospital contacted them to assess them inside the hospital’s facilities.

The sample with AUD was composed of 114 women and 234 men, aged between 27 and 75 years old (*X* = 52.71; *SD* = 9.01). Patients had mostly compulsory education (38.79%) or higher education (34.20%). Nearly 39.66% of participants with AUD were active workers (See Table 1).

**Table 1:** Clinical and sociodemographic description.

Sociodemographic data		AUD group			Control group		
		N/Frequency	Mean (SD)/	Min/Max	N/Frequency	Mean (SD)/	Min/Max
			Frequency (%)			Frequency (%)	
Age		348	52.71 (9.01)	27–75	171	51.92 (8.71)	29–75
Gender	Men	234	67.24%		120	70.18%	
	Women	114	32.76%		51	29.82%	
Educational level	Compulsory education	135	38.79%		43	25.15%	
	High school education / Vocational training	94	27.01%		63	36.84%	
	Superior training	119	34.20%		65	38.01%	
Work situation	Active worker	138	39.66%		141	82.46%	
	Unemployed	73	20.98%		18	10.53%	
	Sick leave	41	11.78%		4	2.34%	
	Student	3	0.86%		0	0.00%	
	Retired	92	26.44%		8	4.68%	
Marital status	Single	75	21.55%		19	11.11%	
	Married	158	45.40%		122	71.35%	
	Divorced	58	16.67%		15	8.77%	
	Separated	13	3.74%		0	0.00%	
	In a relationship	39	11.21%		15	8.77%	
	Widower/Widow	5	1.44%		0	0.00%	

Clinical data							
		AUD group			Control group		
		*N*/Frequency	Mean (SD)/	Min/Max	*N*/Frequency	Mean (SD)/Frequency (%)	Min/Max
			Frequency (%)				
Alcohol intake (yes/no)	Yes	0	0%		117	75.48%	
Abstinence time (in years)		348	3.84 (4.44)	0.8–28	–	–	
Age of initial consumption		348	14.55 (4.07)	4–47	171	16.36 (2.73)	9–72
Age of onset of daily consumption		348	27.89 (10.90)	12–65			
Age of dependence onset		348	29.95 (11.23)	12–65			
Amount of years of dependence		348	22.54 (11.51)	1–52			
Tobacco use	No	173	49.71%		135	78.95%	
	Yes	172	49.43%		36	21.05%	
Other substance use (In the past)	No	206	59.20%		–	–	
	Yes	142	40.80%		–	–	

Clinical and demographic means, standard deviations (SD), and frequency values (expressed in %) for participants with alcohol use disorder (AUD) and control groups.

**Figure 1:**
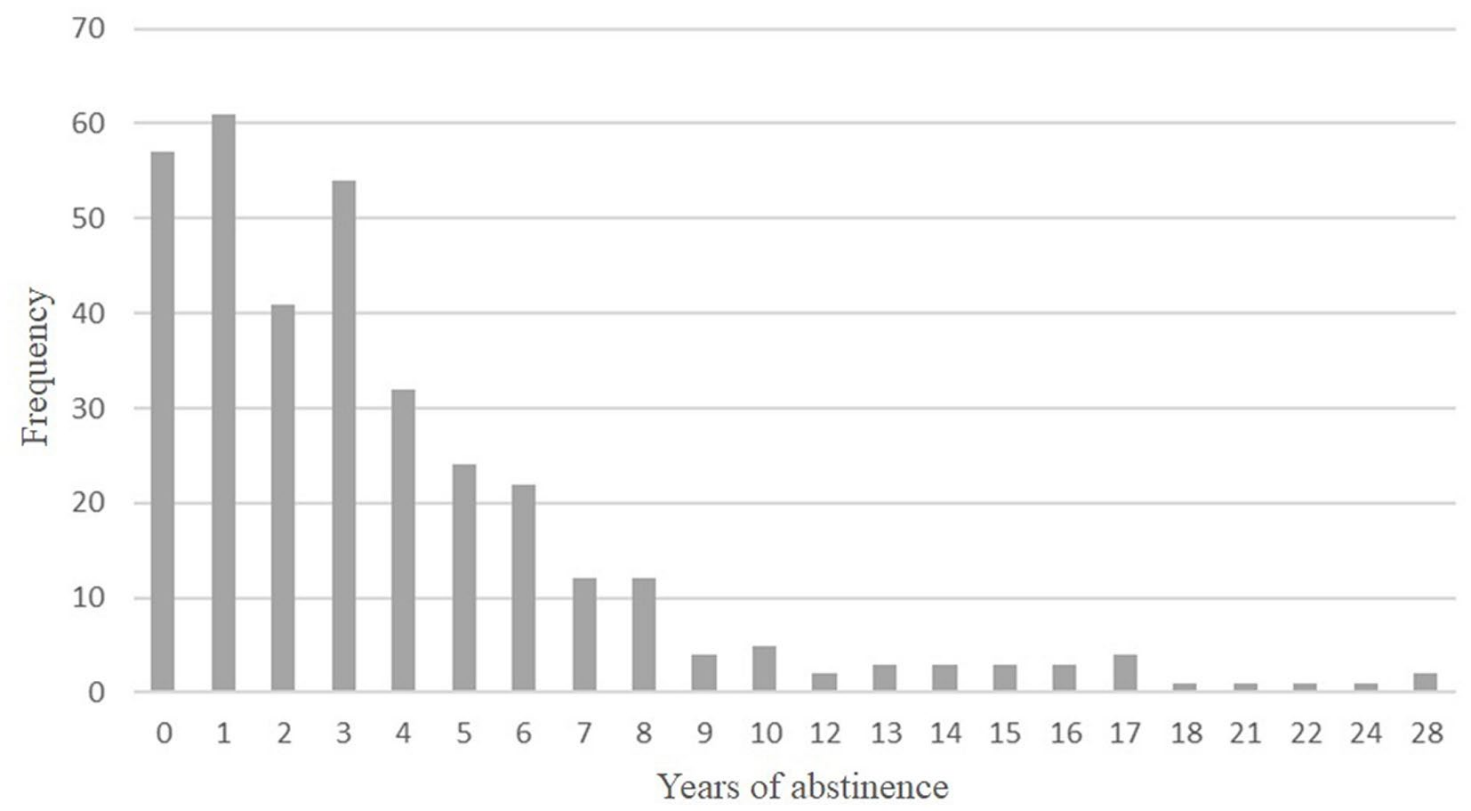
Patients with AUD frequency (expressed in percentages %) distribution according to abstinence time (in years).

Regarding clinical variables (Table 1), the sample was composed of abstinent individuals that had ceased drinking from 1 month to 28 years (*X* = 3.84 years) (see distribution in Figure 1). On average, patients started drinking alcohol during their adolescence (*X* = 14.55; SD = 4.07) and the mean age onset of dependence was approximately 30 years (*X* = 29.95; SD = 11.23). A total of 71.8% of the clinical sample had previous unsuccessful attempts to maintain alcohol abstinence. The number of previous abstinence attempts ranged from 0 to 15, with a mean of 2.11 (SD = 2.71). Most patients had received previous treatments (68.6%). Moreover, 68.6% of the sample showed a family history of substance dependence in first-degree relatives.

All patients were at least 1 month abstinent and had no active consumption of other substances (except for coffee and/or tobacco). Participants diagnosed with any psychiatric or neurological condition were excluded, due to their possible interfering role in the assessments.

With respect to the control group, the sample was composed of 171 participants (120 men and 51 women) with a mean age of 52 years. Nearly 38% of the participants had superior studies (See Table 1). The control group was recruited through a convenience sampling method in the waiting room of several health centers in the local area. Once they manifested interest in the study, the assessor from the 12 de Octubre Psychiatry Service established contact with them and carried out the MINI clinical interview (29) to exclude psychopathologies. All assessments were carried out in the healthcare center’s facilities. Inclusion criteria were also determined by sociodemographic characteristics in an attempt to match control subjects and the AUD group in gender, age, and educational level. Exclusion criteria implied participants diagnosed with substance use disorders or any psychiatric [tested by the international neuropsychiatric interview; MINI (29)] or neurological condition.

### 2.3. Instruments

All scales were administered in their Spanish version.

#### 2.3.1. Psychological scales applied to AUD and control groups

- *Quality of Life Scale (WHOQOL-BREF).* WHOQOL-BREF is a shortened version (27 items) of the original WHOQOL-100 (30). It includes four domains: physical health, psychological health, social relations, and environment, that show an alfa internal consistency of 0.82, 0.81, 0.68, and 0.80, respectively (31). The Spanish version of WHOQOL-BREF shows an internal consistency that ranges between 0.69 and 0.77 (32).
- *Psychological Wellbeing Scale (PWBS).* This scale is based on Ryff’s multidimensional model of psychological wellbeing (33, 34). A version of 54 items (nine by each domain, with six answer options) was applied in the study (35). It comprises six dimensions: self-acceptance, autonomy, environmental mastery, purpose in life, personal growth, and social relations, that show internal consistencies of 0.83, 0.78, 0.77, 0.73, 0.65, and 0.80, respectively (35). The Spanish version, validated in the elderly population (36), has an internal consistency that varies between 0.58 and 0.71.
- *Satisfaction with Life Scale (SWLS).* Satisfaction with life refers to the global assessment that individuals make of their life, by comparing their own circumstances with the vision of what is considered as generally adequate by standard norms (37). The original version shows an internal consistency of 0.87 (37, 38). The Spanish version of SWLS shows an internal consistency of 0.88 (39).
- *Hamilton Anxiety Rating Scale (HAM-A).* A self-informed measure that assesses the severity or intensity of anxiety-like symptoms. It consists of 14 elements defined by symptoms for both psychological and somatic symptoms. Its internal consistency values range between 0.79 and 0.86 (40). It has been translated into Cantonese, French, and Spanish (41). It is the Spanish version of Lobo et al. (42).
- *Hamilton Depression Rating Scale (HAM-D).* A self-informed measure that measures the symptomatic profile and severity of depression. The version used has 21 items with five answer options. Internal consistency values vary between 0.76 and 0.92 (43). The Spanish version of HAM-D shows an internal consistency of 0.78 (44).
- *Barratt Impulsivity Scale (BIS-11).* It evaluates trait impulsivity and behavior through three subscales: motor, cognitive, and non-planned impulsivity. It shows internal consistency values between 0.69 and 0.83 (45). The Spanish version showed adequate linguistic equivalence, conceptual equivalence, and scale equivalence with the original version (46).
- *Acceptance and Action Questionnaire (AAQ-II).* It assesses experiential avoidance and psychological inflexibility. In this study, a Spanish version of 10 items with 7 Likert options was employed. The original scale presents an internal consistency of 0.87 (47).

#### 2.3.2. Psychological scales applied to the AUD group

- *Litman’s Coping Behaviors Inventory (CBI).* It identifies coping strategies employed in order to avoid consumption when experiencing drinking desire or risk-related situations. It distinguishes between four factors or strategies: (A) positive thinking, (B) negative thinking, (C) distraction, and (D) avoiding. These explain 54% of the variance, with coefficients of 0.91, 0.81, 0.65, and 0.75, respectively (48). Studies in Spanish samples show internal consistencies of 0.90 in alcohol-dependent individuals (49).
- *Recovery Capital Assessment (VCR).* It evaluates 10 elements involved in recovery: abstinence/use of substances, global psychological health, global physical health, community involvement, social support, leisure activities, family environment, risk taking, life functioning, and recovery experience. The original one-dimensional scale shows intraclass correlations between 0.50 and 0.73 (50), and the Spanish adaptation shows an internal consistency of 0.90 (51).

#### 2.3.3. Psychological scales applied to the control group

- *Mini-International Neuropsychiatric Interview (MINI).* The MINI is a short-structured diagnostic interview compatible with DSM-III-R/IV and ICD-10 criteria (52). The instrument included a series of questions about the following symptoms: sleep, feeding, depression, panic attacks, generalized anxiety disorder, obsessive–compulsive disorder, post-traumatic stress disorder (PTSD), suicidal risk, substance abuse disorder (SAD), and cognitive complaints. This study used the Spanish version of the interview validated by Ferrando (29).
- *Alcohol Use Disorders Inventory Test (AUDIT):* It is a 10-item self-administered questionnaire which covers the domains of alcohol consumption, drinking behavior, and alcohol-related problems. Responses to each question are scored from 0 to 4, giving a maximum possible score of 40 (53). It is recommended by the WHO (World Health Organization) as a screening test (53). It is one of the most widely used worldwide, both in healthcare and non-healthcare settings. Internal consistency (Cronbach *α*) values are 0.80 for the controls and 0.80 for the alcohol-dependent individuals (54). It has a Spanish validation carried out by Rubio et al. (55).

### 2.4. Statistical analysis

An exploratory analysis was carried out to evaluate sociodemographic, clinical, and psychological dimensions. AUD and control groups were analyzed in descriptive terms (mean and standard deviations) for continuous variables and by frequencies for categorical ones.

Continuous variables were submitted through normality tests by the Kolmogorov–Smirnov index. Intergroup comparisons were performed for AUD versus control groups and according to gender by parametrical Student *t*-tests if they followed a normal distribution or the Mann–Whitney–Wilcoxon test if not. Adjusted value of *p*s for multiple comparisons were realized using the Benjamini and Hochberg (56) method. Categorical data were compared through the chi-square test (χ^2^). The significance level was set at a value of *p* of <0.05.

Psychological dimensions were evaluated as a function of abstinence time, through regression model analyses. Before this step, psychological test scores were normalized to *Z* scores. All models were evaluated by the need to adjust to the non-linear presence of years of abstinence, as well as its squared and cubic values, in addition to the linear component of years of abstinence. The starting point was the saturated model with non-transformed years of abstinence and the quadratic and cubic transformations, included as variables in the same model, in addition to the variables age and gender. The selection of the best model was carried out using the Akaike information criterion (AIC). Regardless of statistical significance, age and gender were incorporated into the model as covariates. For each explanatory model, the estimated value for an individual can be estimated by substituting the subject values into the equation: β0 + β1*age + β2* gender +β3*(years abstinent) + β4*(years abstinent)2 + β5*(years abstinent)3. β0 indicates the starting level of the dependent variable for a person at abstinence time 0, aged 52.71 years and male. The value of the dependent variable will change β1 times for every unit change in the person’s age relative to the mean age of 52.71 years, a quantity of β2 as a function of being a female relative to being a male, and β3 times in combination with β4 and β5 for every unit increase in the abstinence time variable. It should be noted that in some models, quadratic or cubic years of abstinence were discarded. In those cases, where the AIC discarded the non-transformed abstinence time variable because it was not statistically significant, the regression model graphs were built with the average of the following variables: gender, age, and β0.

Scatter plots were used to explore inflection points and scores matching between the AUD group and the control group. Regression plots show the intersection between point estimations of the AUD equation line and the average scores for control subjects.

All data were inserted and analyzed by SPSS v.22 (57) and SAS v.9.4 (58).

## 3. Results

### 3.1. Intergroup comparison of psychological dimensions (*t*-test AUD group vs. control group)

Table 2 includes the descriptive data for the different psychological scales applied to AUD and control subjects. It also includes the mean comparisons of the scores of different scales between the AUD and control groups, using Student’s *t*-test. The *t*-test shows that there are statistically significant differences (*p* < 0.05) between control and AUD groups for the following scales and subscales: social relations (WHOQOL); all the PWBS subscales (except for personal growth); satisfaction with life (SWLS); depression (HAM-D); anxiety (HAM-A); impulsivity total score (BIS-11); and experiential avoidance and psychological inflexibility (AAQ-II).

**Table 2:** Psychological measures description and Student T intergroup comparisons.

	AUD group			Control group			Student *T* comparisons		
Variables	Mean (SD)	Min.	Max.	Mean (SD)	Min.	Max.	*t*	df	*p*
Quality of Life (WHOQOL-BREF)									
Physical health	13.67 (1.88)	8.00	17.71	13.50 (1.45)	9.71	18.29	1.097	424.291	0.273
Psychological health	14.04 (2.25)	6.67	18.00	14.20 (1.63)	9.33	17.33	−0.928	445.762	0.354
Social relations	13.56 (3.01)	5.00	20.00	14.48 (3.00)	6.67	20.00	−3.283	513	0.001
Environment	15.40 (2.08)	9.00	20.00	15.09 (1.92)	10.50	20.00	1.627	513	0.104
Psychological Wellbeing (PWBS) and Satisfaction with Life (SWLS)									
Autonomy	37.60 (6.68)	17.00	51.00	39.76 (6.30)	23.00	54.00	−3.526	517	<0.001
Relations with others	39.87 (7.73)	20.00	54.00	41.78 (6.19)	25.00	54.00	−3.030	411.539	0.003
Self-Acceptance	35.61 (8.29)	10.00	53.00	40.57 (6.35)	23.00	53.00	−7.510	427.544	<0.001
Environmental Mastery	38.70 (8.22)	16.00	54.00	41.20 (6.44)	18.00	53.00	−3.760	421.686	<0.001
Purpose in Life	37.05 (7.52)	13.00	54.00	39.56 (5.58)	27.00	51.00	−3.858	509	<0.001
Personal Growth	37.66 (7.96)	15.00	53.00	37.94 (6.98)	26.00	52.00	−0.394	506	0.694
Satisfaction With Life	21.33 (6.47)	5.00	35.00	24.14 (3.52)	17.00	33.00	−6.397	508.667	<0.001
Negative emotionality									
Depression (HAM-D)	11.76 (8.15)	1.00	47.00	5.40 (5.42)	0.00	28.00	10.566	472.176	<0.001
Anxiety (HAM-A)	9.03 (6.85)	1.00	42.00	3.44 (4.80)	0.00	36.00	10.757	456.168	<0.001
Impulsivity. Total Score (BIS-11)	45.41 (14.75)	8.00	84.00	36.88 (11.02)	11.00	73.00	7.349	436.765	<0.001
Experiential avoidance and psychological inflexibility (AAQ-II)	32.37 (11.14)	11.00	64.00	24.54 (8.26)	10.00	45.00	9.006	438.443	<0.001
Coping strategies (CBI)									
Positive thinking	28.90 (4.53)	10.00	37.00	–	–	–	–	–	–
Negative thinking	17.34 (5.39)	0.00	24.00	–	–	–	–	–	–
Distraction	16.89 (6.66)	0.00	30.00	–	–	–	–	–	–
Avoidance	9.03 (3.06)	0.00	15.00	–	–	–	–	–	–
Other measures									
Recovery Capital	43.55 (6.68)	8.00	50.00	–	–		–	–	–
AUDIT	–	–	–	3.13 (2.83)	0.00	11.00	–	–	–

Psychological measures descriptions in terms of means, standard deviations (SD), and minimum (Min.) and maximum (Max.) scores for participants with alcohol use disorder (AUD) and control subjects. Student T comparisons are also presented, through t-index, degree of freedom (df), and value of *p*s.

### 3.2. Gender differences in the AUD group

Compared to men with AUD, women obtained significantly lower scores in WHOQOL-BREF’s physical health subscale (*t* = −2.46; *p* = 0.014). On the counterpart, they showed significantly higher scores than men in Hamilton’s depression (*t* = 2.64; *p* = 0.009), anxiety scales (*t* = 4.30; *p* = 0.000), and experiential avoidance one (*t* = 3.20; *p* = 0.002). Women also had significantly higher scores in motor impulsivity measured by BIS-11 (*t* = 2.16; *p* = 0.032).

### 3.3. Psychological dimensions as a function of abstinence time

#### 3.3.1. Quality of life (WHOQOL-BREF) and recovery capital

**Figure 2:**
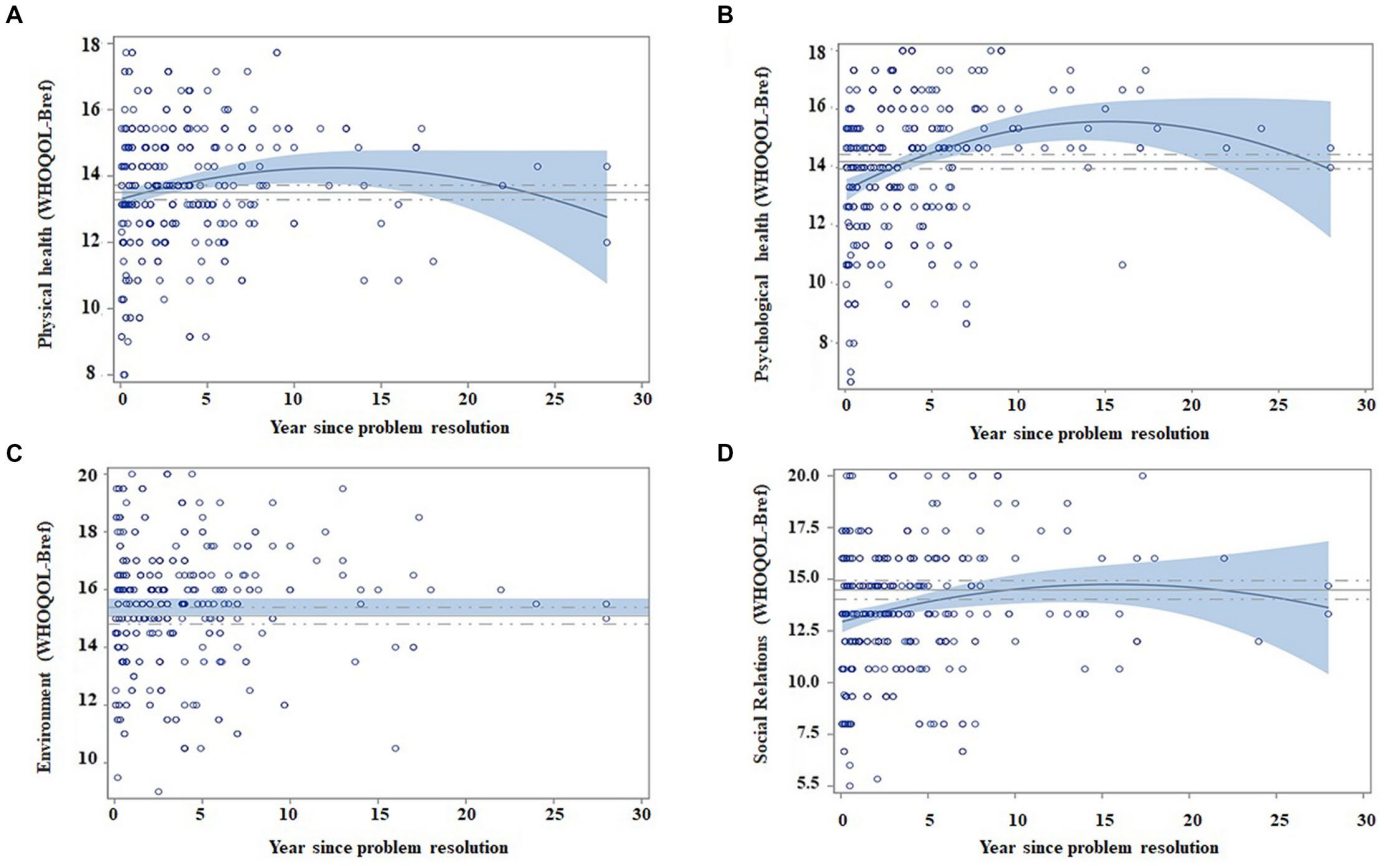
Regression models of quality of life as a function of abstinence time, adjusted by age and gender. Matching AUD with controls. Scatter plots indicate the recovery indexes (standardized) during the first 28 years after ceasing alcohol consumption. For each explicative model, the estimated value for an individual can be estimated by displacing the subject’s values in the following equation: β0 + β1 * age + β2 * gender + β3 * (years of abstinence) + β4 * (years of abstinence)^2^. The continuous blue line indicates the regression equation between abstinence time and the scores obtained from the questionnaires. The blue shading represents the confidence interval (95%) for the regression equation in the AUD group. The continuous gray line indicates the mean score obtained on each questionnaire by the control subjects, and the dashed gray line indicates the confidence interval (95%). The figure depicts the following: **(A)** Patients improve their scores in physical health over time (Year since problem resolution), and they match control scores at 1 year of abstinence; **(B)** patients improve in psychological health over time and match control values at the 4th year of abstinence; **(C)** the environment dimension does not show changes, and it seems to be similar between patients and controls; and **(D)** social relations increase after ceasing alcohol consumption and equate control scores at 10 years of abstinence.

Scatter plots in Figure 2 show the regression models for standardized scores in quality of life, in relation to abstinence time (transformed to quadratic values for physical health, psychological health, and relations). Regarding quality-of-life dimensions, physical health seems to improve faster, followed by psychological health and social relations, with negligible changes in the environment.

The regression model did not identify abstinence time as a variable that would contribute to WHOQOL-BREF environment variability (see Table 3). The remaining WHOQOL-BREF’s dimensions (physical, psychological health, and social relations) improve with abstinence time, regardless of the age and gender of subjects, except for physical health scores, that were higher for male subjects (see Table 3).

**Table 3:** Regression model for quality of life and recovery capital as a function of abstinence time, adjusted by age and gender in the AUD group.

Model	Beta	SE	*t*	Value of *p*
Quality of Life (WHOQOL-BREF)				
WHOQOL-Physical Health				
intercept	13.511	0.184	73.500	<0.0001
Age	0.017	0.012	1.430	0.152
Gender	−0.447	0.216	−2.060	0.040
Years	0.134	0.055	2.430	0.016
Years (quadratic)	−0.006	0.003	−2.270	0.024
WHOQOL-psychological health				
intercept	13.179	0.216	61.150	<0.0001
Age	0.014	0.014	1.010	0.313
Gender	0.201	0.254	0.790	0.430
Years	0.301	0.065	4.640	<0.0001
Years (quadratic)	−0.010	0.003	−3.170	0.002
WHOQOL-social relations				
intercept	13.071	0.296	44.120	<0.0001
Age	0.026	0.019	1.390	0.165
Gender	−0.190	0.349	−0.540	0.586
Years	0.210	0.089	2.360	0.019
Years (quadratic)	−0.007	0.004	−1.680	0.093
WHOQOL-environment				
intercept	15.382	0.137	112.300	<0.0001
Age	0.028	0.013	2.230	0.026
Gender	0.064	0.242	0.260	0.793
VCR-recovery capital				
intercept	41.200	0.642	64.20	<0.0001
Age	0.079	0.042	1.90	0.059
Gender	1.223	0.763	1.60	0.110
Years	0.694	0.192	3.61	0.0004
Years (quadratic)	−0.021	0.009	−2.27	0.024

Table expresses regression models for quality of life (WHOQOL-BREF) and recovery capital (VCR) as a function of abstinence time, adjusted by age and gender. Beta, *SE* (standard error), *t*, and value of *p*s are indicated for each type of variable. For each model, the estimated value for an individual can be computed by replacing his values from the equation: β0 + β1*age + β2*gender + β3*(years of abstinence) + β4*(years of abstinence)^2^. All models were evaluated and adjusted for the non-linear presence of years of abstinence as a raw value, quadratic or cubic estimate, as well as for the linear component of abstinence. Generally, β0 indicates the starting point for quality of life or recovery capital for an individual at time of abstinence of value 0, for a male, of 52.71 years old.

When matching AUD participants and control subjects, we observed that patients reached similar scores (with an interval confidence of 95%) in the 1st year of abstinence for physical health, the 4th year for psychological health, and the 10th year for relations with others (Figure 2). Despite the fact that environment does not vary with abstinence time, its scores were similar between patients and control subjects (*p* = 0.104), as observed in the *t*-test AUD group versus control group section.

With respect to recovery capital, AIC selected the quadratic model. The plateau of the regression curve began at the 10th year of abstinence (see Table 3).

#### 3.3.2. Psychological wellbeing and satisfaction with life

**Figure 3:**
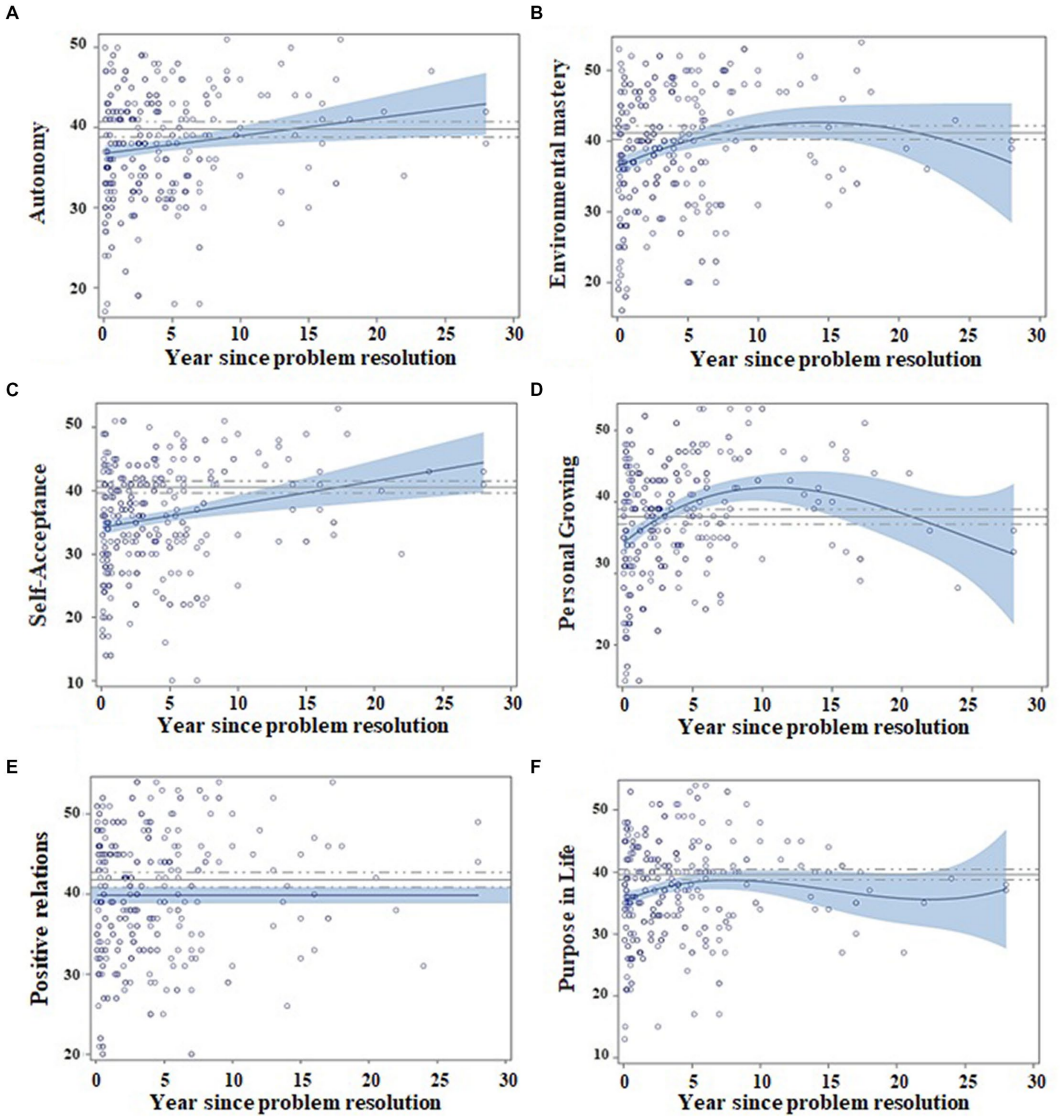
Regression models for psychological wellbeing as a function of abstinence time, adjusted by age and gender. Matching AUD with controls. Scatter plots indicate the recovery indexes (standardized) during the first 28 years after ceasing alcohol consumption. For each explicative model, the estimated value for an individual can be estimated by displacing the subject’s values in the following equation: β0 + β1 * age + β2 * gender + β3 * (years of abstinence) + β4 * (years of abstinence)^2^ + β5 * (years of abstinence)^3^. The continuous blue line indicates the regression equation between abstinence time and the scores obtained from the questionnaires. The blue shading represents the confidence interval (95%) for the regression equation in the AUD group. The continuous gray line indicates the mean score obtained on each questionnaire by the control subjects, and the dashed gray line indicates the confidence interval (95%). The figure depicts the following: **(A)** Patients improve their scores in autonomy over time (year since problem resolution), and they match control scores at 15 years of abstinence; **(B)** patients improve in environmental mastery over time and match control values at the 7th year of abstinence; **(C)** the self-acceptance dimension is better in time and AUD patients match control scores at 22 years of abstinence; **(D)** personal growing AUD scores overcome controls and reach a maximum at 10 years, showing a decrease afterward; **(E)** relations with others do not show changes over time; and **(F)** purpose in life grows over time and matches control values at 10 years of abstinence.

**Figure 4:**
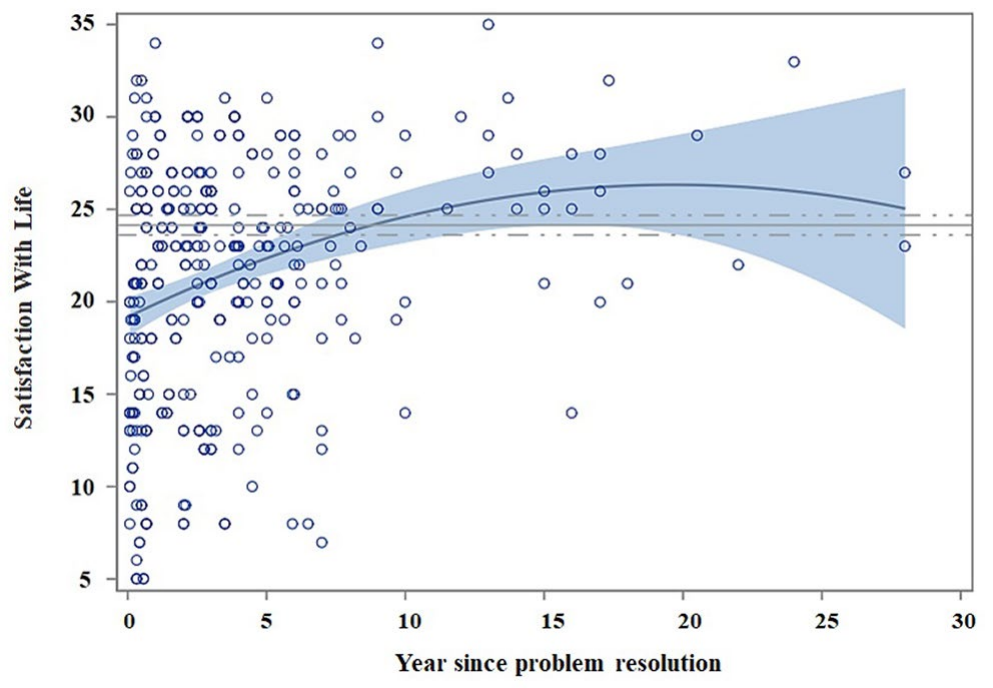
Regression model for satisfaction with life as a function of abstinence time. Matching AUD with controls. Scatter plots indicate the recovery indexes (standardized) during the first 28 years after ceasing alcohol consumption. For each explicative model, the estimated value for an individual can be estimated by displacing the subject’s values in the following equation: β0 + β1 * age + β2 * gender + β3 * (years of abstinence) + β4 * (years of abstinence)^2^. The continuous blue line indicates the regression equation between abstinence time and the scores obtained from the questionnaires. The blue shading represents the confidence interval (95%) for the regression equation in the AUD group. The continuous gray line indicates the mean score obtained on each questionnaire by the control subjects, and the dashed gray line indicates the confidence interval (95%). The scatter plot indicates the improvement in satisfaction with life over time (year since problem resolution) and the equation with control subjects at 10 years of abstinence.

Scatter plots in Figures 3, 4 show regression models for PWBS and SWLS standardized scores in relation to abstinence time. The fastest changes (in the first 4–5 years) occur in environmental mastery, personal growth, purpose in life, and satisfaction with life, whereas the slowest ones take place for self-acceptance and autonomy. Additionally, no clear changes were appreciated for positive relations.

As Table 4 shows, Psychological Wellbeing and Life Satisfaction scores increase as abstinence is maintained for both men and women. However, the regression model did not find abstinence time as a variable with an effect on positive relations (patients and control subjects showed significantly different scores, *p* = 0.005, see section *t*-test AUD group vs. control group). Additionally, age was a factor that increased PWBS and SWLS scores, except for the purpose in life and personal growing PWBS subscales.

**Table 4:** Regression model for psychological wellbeing and satisfaction with life as a function of abstinence time, adjusted by age and gender in the AUD group.

Model	Beta	SE	*t*	Value of *p*
Psychological wellbeing (PWBS)				
PWBS-autonomy				
Intercept	36.885	0.552	66.870	<0.0001
Age	0.111	0.041	2.680	0.008
Gender	0.301	0.766	0.390	0.695
Years	0.161	0.083	1.930	0.055
PWBS-positive relations				
Intercept	39.472	0.503	78.450	<0.0001
Age	0.131	0.046	2.820	0.005
Gender	1.218	0.889	1.370	0.171
PWBS-self-acceptance				
Intercept	34.807	0.679	51.280	<0.0001
Age	0.141	0.052	2.730	0.007
Gender	−0.832	0.946	−0.880	0.380
Years	0.272	0.103	2.650	0.009
PWBS-environmental mastery				
Intercept	36.496	0.802	45.480	<0.0001
Age	0.149	0.051	2.900	0.004
Gender	0.758	0.943	0.800	0.422
Years	0.774	0.240	3.220	0.001
Years (quadratic)	−0.029	0.012	−2.520	0.012
PWBS-purpose in life				
Intercept	35.092	0.852	41.160	<0.0001
Age	−0.072	0.048	−1.500	0.134
Gender	−0.231	0.885	−0.260	0.794
Years	1.115	0.416	2.680	0.008
Years (quadratic)	−0.096	0.048	−2.020	0.044
Years (cubic)	0.002	0.001	1.600	0.111
PWBS-personal growth				
Intercept	35.092	0.852	41.160	<0.0001
Age	−0.072	0.048	−1.500	0.134
Gender	−0.231	0.885	−0.260	0.794
Years	1.115	0.416	2.680	0.008
Years (quadratic)	−0.096	0.048	−2.020	0.044
Years (cubic)	0.002	0.001	1.600	0.111
SWLS-satisfaction with life				
Intercept	19.174	0.612	31.340	<0.0001
Age	0.091	0.039	2.340	0.020
Gender	0.582	0.721	0.810	0.421
Years	0.671	0.183	3.660	<0.0001
Years (quadratic)	−0.018	0.009	−1.990	0.047

Table expresses regression models for psychological wellbeing (PWBS) and satisfaction with life (SWLS) as a function of abstinence time, adjusted by age and gender. Beta, *SE* (standard error), *t*, and value of *p*s are indicated for each type of variable. For each model, the estimated value for an individual can be computed by replacing his values from the equation: β0 + β1*age + β2*gender + β3*(years of abstinence) + β4*(years of abstinence)^2^ + β5*(years of abstinence)^3^. All models were evaluated and adjusted for the non-linear presence of years of abstinence as a raw value, quadratic or cubic estimate, as well as for the linear component of abstinence. Generally, β0 indicates the starting point for psychological wellbeing and satisfaction with life for an individual at time of abstinence of value 0, for a male, of 52.71 years old.

Figures 3, 4 show that patients match controls in environmental mastery at 7 years of abstinence, in purpose in life, personal growth and satisfaction with life at 10 years, in autonomy at 15 years, and for self-acceptance at 22 years. AUD participants did not match control scores for positive relations at any measured abstinence period.

#### 3.3.3. Affective and impulsivity manifestations

**Figure 5:**
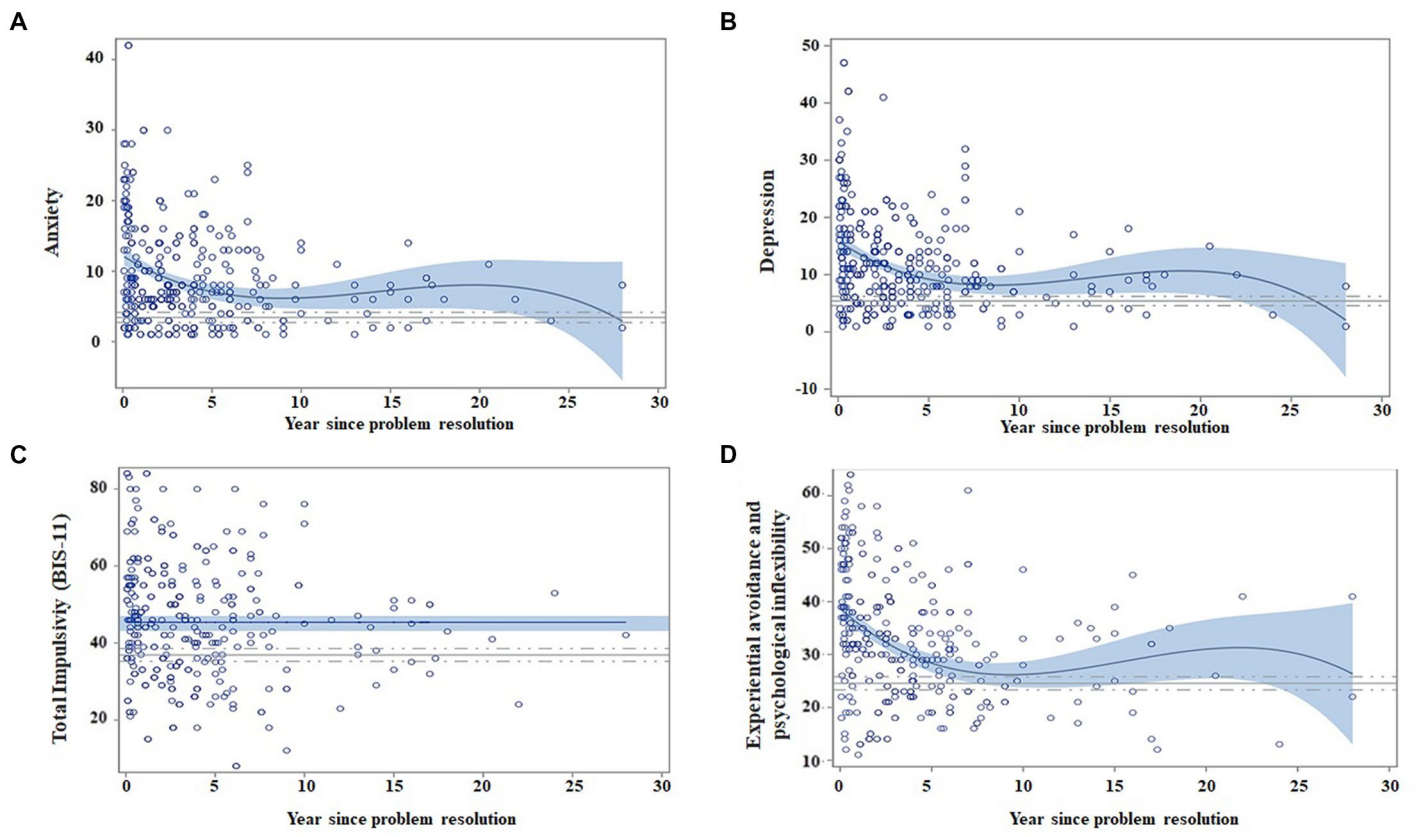
Regression models for negative emotionality and impulsivity dimensions, as a function of abstinence time, adjusted by age and gender. Matching AUD with controls. Scatter plots indicate the recovery indexes (standardized) during the first 28 years after ceasing alcohol consumption. For each explicative model, the estimated value for an individual can be estimated by displacing the subject’s values in the following equation: β0 + β1 * age + β2 * gender + β3 * (years of abstinence) + β4 * (years of abstinence)^2^ + β5 * (years of abstinence)^3^. The continuous blue line indicates the regression equation between abstinence time and the scores obtained from the questionnaires. The blue shading represents the confidence interval (95%) for the regression equation in the AUD group. The continuous gray line indicates the mean score obtained on each questionnaire by the control subjects, and the dashed gray line indicates the confidence interval (95%). The figure depicts the following: **(A)** Patients have less anxiety over time (year since problem resolution), and they match control scores at 10 years of abstinence (fluctuations are due to the small sample size); **(B)** depression also diminishes over time over time and match control values at the 7th year of abstinence; **(C)** total impulsivity does not show changes over time; and **(D)** experiential avoidance and psychological inflexibility decrease over time and match control values at 10 years of abstinence.

Scores in anxiety, depression, and experiential avoidance progressively declined across the first 5–7 years, and then, they slowly diminished (non-linear regression model representation can be seen in Figure 5, and its data can be checked in Table 5). The regression model did not identify abstinence time as a variable that would contribute to impulsivity variability (Table 5).

**Table 5:** Regression model for negative emotionality and impulsivity as a function of abstinence time, adjusted by age and gender.

Model	Beta	SE	*t*	*p*-value
Hamilton anxiety (*)				
Intercept	11.040	0.730	15.13	<0.0001
Age	−0.012	0.041	−0.30	0.764
Gender	2.905	0.753	3.86	0.0001
Years	−1.542	0.354	−4.35	<0.0001
Years (quadratic)	0.129	0.041	3.17	0.002
Years (cubic)	−0.003	0.001	−2.57	0.011
Hamilton depression				
Intercept	14.873	0.866	17.18	<0.0001
Age	−0.115	0.049	−2.37	0.018
Gender	1.633	0.894	1.83	0.069
Years	−1.938	0.420	−4.61	<0.0001
Years (quadratic)	0.168	0.048	3.47	0.001
Years (cubic)	−0.004	0.001	−2.89	0.004
BIS-11-Total. Impulsivity				
Intercept	44.986	0.957	47.01	<0.0001
Age	−0.348	0.088	−3.93	0.0001
Gender	1.173	1.694	0.69	0.489
AAQ-II. Experiential avoidance and psychological inflexibility				
intercept	37.030	1.157	32.01	<0.0001
Age	−0.182	0.065	−2.81	0.005
Gender	2.838	1.194	2.38	0.018
Years	−2.854	0.561	−5.08	<0.0001
Years (quadratic)	0.223	0.065	3.45	0.001
Years (cubic)	−0.005	0.002	−2.51	0.012

Table expresses regression models for Hamilton Anxiety and Depression variables as well as Impulsivity (BIS-11) and experiential avoidance and psychological inflexibility (AAQ-II), as a function of abstinence time, adjusted by age and gender. Beta, *SE* (standard error), *t*, and *p*-values are indicated for each type of variable. For each model, the estimated value for an individual can be computed by replacing his values from the equation: β0 + β1*age + β2*gender + β3*(years of abstinence) + β4*(years of abstinence)^2^ + β5*(years of abstinence)^3^. All models were evaluated and adjusted for the non-linear presence of years of abstinence as a raw value, quadratic or cubic estimate, as well as for the linear component of abstinence. Generally, β0 indicates the starting point for affective symptoms (anxiety and/or depression) or experiential avoidance for an individual at time of abstinence of value 0, for a male, of 52.71 years old. Abstinence time does not influence BIS-11 values since it is does not appear in the equation.

Regression models from Table 5 indicate that all negative emotionality dimensions diminished with abstinence maintenance, regardless of age, whereas being a woman was associated with higher scores in anxiety and experiential avoidance.

Figure 5 shows that patients reached similar scores to control subjects after 7 years of abstinence for depression and at 10 years of abstinence for anxiety and experiential avoidance. AUD patients did not match control scores for impulsivity (as seen in the *t*-test AUD group vs. control group, there are statistically significant differences; *p* = 0.001).

#### 3.3.4. Coping strategies against alcohol use (CBI)

**Figure 6:**
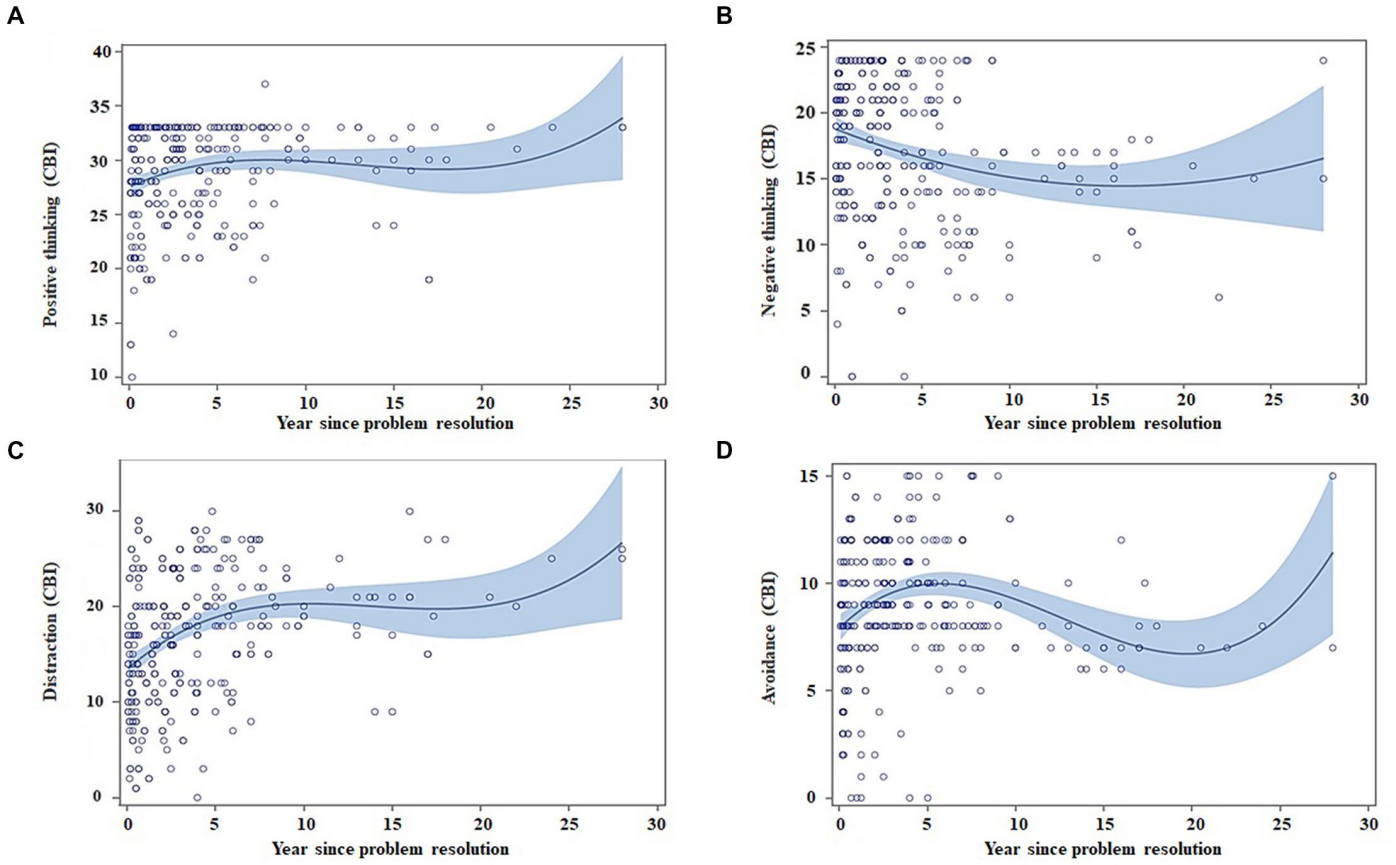
Regression models for coping strategies (CBI) as a function of abstinence time, adjusted by age and gender. Scatter plots indicate the recovery indexes (standardized) during the first 28 years after ceasing alcohol consumption. For each explicative model, the estimated value for an individual can be estimated by displacing the subject’s values in the following equation: β0 + β1 * age + β2 * gender + β3 * (years of abstinence) + β4 * (years of abstinence)^2^ + β5 * (years of abstinence)^3^. The blue shading represents the confidence interval (95%) for the regression equation in the AUD group. **(A)** Patients show an increase in the use of the positive thinking throughout the time of abstinence **(B)** the use of the negative thinking strategy decreases throughout the time of abstinence; **(C)** the use of the distraction strategy increases throughout the time of abstinence; and **(D)** the use of the avoidance strategy increases in the first years of abstinence and subsequently decreases.

Coping strategies differed in their evolution throughout the abstinence maintenance. Figure 6 graphs show an improvement in all subscales in the first 5 years and posterior stabilization.

Regarding CBI, subscale changes across abstinence periods, positive and negative thinking, and distraction adjusted to a non-linear model, while avoidance had a quadratic model (Table 6). Negative thinking scores decreased along the abstinence, whereas the rest of coping strategies increased. Specifically, avoidance strategies increased during the 1st year and then had light stabilization and a posterior decrease. Changes occurred irrespectively of age, except for positive thinking.

**Table 6:** Regression model for coping strategies as a function of abstinence time, adjusted by age and gender.

Model	Beta	SE	*t*	*p*-value
Positive thinking				
Intercept	27.362	0.509	53.78	<0.0001
Age	0.069	0.029	2.43	0.016
Gender	1.197	0.543	2.21	0.028
Years	0.645	0.250	2.58	0.010
Years (quadratic)	−0.060	0.028	−2.10	0.036
Years (cubic)	0.002	0.001	1.93	0.055
Negative thinking				
Intercept	18.775	0.530	35.41	<0.0001
Age	−0.005	0.034	−0.14	0.891
Gender	0.075	0.645	0.12	0.908
Years	−0.522	0.160	−3.25	0.001
Years (quadratic)	0.016	0.008	2.04	0.043
Distraction				
intercept	12.987	0.721	18.00	<0.0001
Age	0.037	0.040	0.91	0.363
Gender	1.631	0.767	2.13	0.034
Years	1.586	0.355	4.46	<0.0001
Years (quadratic)	−0.119	0.040	−2.96	0.003
Years (cubic)	0.003	0.001	2.48	0.014
Avoidance				
intercept	7.809	0.342	22.82	<0.0001
Age	−0.025	0.019	−1.32	0.189
Gender	0.024	0.363	0.07	0.947
Years	0.839	0.168	4.98	<0.0001
Years (quadratic)	−0.093	0.019	−4.86	<0.0001
Years (cubic)	0.002	0.001	4.49	<0.0001

Table expresses regression models for coping strategies against alcohol consumption variables, as a function of abstinence time, adjusted by age and gender. Beta, *SE* (standard error), *t*, and *p*-values are indicated for each type of variable. For each model, the estimated value for an individual can be computed by replacing his values from the equation: β0 + β1 * age + β2 * gender + β3 * (years of abstinence) + β4 * (years of abstinence)^2^ + β5*(years of abstinence)^3^. All models were evaluated and adjusted for the non-linear presence of years of abstinence as a raw value, quadratic or cubic estimate, as well as for the linear component of abstinence. Generally, β0 indicates the starting point for coping strategies for an individual at time of abstinence of value 0, for a male, of 52.71 years old.

## 4. Discussion

The aim of this study was to examine how, when, and to what extent persons with alcohol use disorder (AUD) recover their psychological wellbeing and quality of life. Most studies regarding AUD recovery had been carried out with samples that had rather short periods of abstinence, impeding the analysis of long-term recovery, changing patterns, or the improvement on several psychological dimensions over time, in comparison with healthy participants (16). This is the first study carried out in a Spanish clinical sample of alcohol-dependent individuals with different abstinence periods and a healthy control group.

The main findings of this study were as follows: (A) Recovery is a long process that may involve a relation between abstinence length and improvements in psychological wellbeing, quality of life, and recovery capital; (B) changes in negative emotionality could also associated to abstinence duration; (C) during the 1st year of abstinence, the prevailing coping strategies were avoidance and distraction, while with further abstinence, positive thinking strategies seemed to increase while avoidance became a less used strategy; (D) the most pronounced changes occurred during the first 5 years in all the psychological dimensions studied; (E) recovery in women with AUD differed from the one developed in men; and (F) patient seemed to reach similar scores to control subjects, according to different abstinence periods: first, in physical health (the 1st year); second, in psychological health (4th year) and the following subscales at longer periods of abstinence, with the exception of two dimensions that remained different from healthy participants: impulsivity and positive relations.

### 4.1. How do psychological dimensions of wellbeing, quality of life, negative emotionality, and coping strategies recover depending on abstinence?

Recovery is a slow process, where long-term abstinence maintenance relates to improvements in quality of life and wellbeing. Abstinence duration is also linked to other dimensions associated with recovery, such as negative emotionality (depression and anxiety symptoms and experiential avoidance) and coping strategies to avoid consumption. The results exhibit more pronounced changes in psychological recovery dimensions during the first 5 years, after ceasing alcohol consumption. Specifically, our findings indicate marked improvements in quality-of-life subscales during the first 4 years of abstinence, with the following sequence relation: physical–psychological–social relations. Two reviews pointed out the decline in quality of life in AUD and its improvement after treatment (24, 59) although most studies were carried out within short or medium abstinence periods (1–18 months). Of note is the study by Frischknecht et al. (60). This study found positive correlations between quality-of-life scores and maintenance of abstinence in alcohol-dependent patients, 7 years after treatment (*r* = 0.316; *p* < 0.01). People who remained abstinent showed better scores than those who kept consuming alcohol (60).

Abstinence periods also relate to enhancements in recovery capital, as well as psychological wellbeing and satisfaction with life. The latter rapidly improved during the first 4–5 years and then attenuated their increase. Furthermore, negative emotionality scores rapidly decreased during the first 5–7 years and attenuated their course over longer periods of abstinence. These results support the biaxial model of recovery of Kelly and Hoeppner (3), which proposes that greater availability and accumulation of recovery capital would favor the resilience and coping strategies and help reduce and buffer the stress, subsequently sustaining continuous remission (3, 61). In this line, psychological wellbeing has been proposed as a protective factor for stress and anger (62), which may reflect a similar mechanism to the one proposed by Kelly and Hoeppner (3). Carlon et al. (63) proposed a similar mechanism for improvements in quality of life. This study suggests that positive and negative affect, as well as decreased stress experiences, help explain why QOL increases significantly for individuals following treatment for AUD (10 weeks, 36 weeks, and 52 weeks following treatment) (63). This suggests that improvements in some areas favor amelioration in others, thus constituting elements of recovery beyond abstinence.

Regarding the coping strategies, to the best of our knowledge, this is the first time they have been evaluated in the context of long-term recovery. Our findings evidence that persons with AUD that follow treatment show a fast enhancement in their coping strategies repertoire, which has the aim to prevent a new consumption (especially during the first 5 years). This would support the proposal of Laudet (64), which presents coping strategies management as one of the pillars of recovery. These results indicate that strategies such as distraction and avoidance are employed more promptly than other cognitive strategies. However, while the use of avoidance is reduced with prolonged abstinence (after 7 years), the use of positive thinking and distraction is maintained through time. Unlike the rest of the strategies, negative thinking diminishes over abstinence time, meaning that reflecting more about the negative consequences of consumption when risky situations take place might not be the most used strategy in order to remain abstinent, whereas reflecting on the benefits of non-consumption, by using distraction strategies and avoiding risky situations are more used through time and might be more beneficial. These precise results regarding strategies that consolidate during the first abstinence periods coincide with our previous studies (27, 28), where avoidance was established as the most solid strategy used to maintain abstinence after a 6-year follow-up. In the same way, the gradual use of positive thinking would be in agreement with Litman’s study that proposes a transition from behavioral to cognitive coping strategies along the maintained abstinence (65). Additionally, the slow yet sustained increase in positive thinking could relate to the changes in wellbeing, as Laudet et al. (64) proposed having something to lose if the consumption is resumed would be one of the strongest individual predictors associated with remission; and the possible losses might occur in the areas of satisfaction with life, health, acquaintances, and family members.

With respect to gender differences, the results indicate that women show more difficulties to recover in physical health and negative emotionality dimensions. These differences have also been pointed out in other studies (16, 66) that indicate that women tend to experience more psychological distress than men. As Kelly et al. (16) proposed, recovery for women could suppose a greater challenge when dealing with psychological stress and lower satisfaction with quality-of-life aspects. In this way, interventional-recovery programs should offer emotional control improvement strategies for women. Nonetheless, general population (non-clinical) results show higher self-perceived health in women compared to men (67–69). Overall, a special consideration toward gender should be implemented in the study of recovery, as other health contexts already attempt.

### 4.2. Matching psychological dimensions with healthy participants: to what extent can patients improve?

Matching psychological dimension scores of AUD participants with the control group happened at different time periods of abstinence. AUD individuals seem to match healthy controls in quality of life in the first 4 years (with the exception of social relations, that happened over 10 years of abstinence) and in psychological wellbeing after more than 10 years of abstinence. This might imply a long course of the recovery process. Additionally, in our samples, some variables never seem to equal the control group, such as positive relations.

Based on quality of life, psychological wellbeing, and coping strategies scores, our results allow to draw a possible staging of the following recovery phases in AUD persons that follow treatment or attend self-help associations (see Table 7). *First Stage/Early Sobriety (0–1 years)*: improvement of physical quality of life, reaching values similar to healthy subjects; great improvement in anxiety; and use of behavioral strategies (such as distraction and avoidance). *Second Stage/Sustained Sobriety (1–4 years)*: enhancement in psychological quality of life, reaching values similar to controls; distinct improvement in affective dimensions such as sadness and experiential avoidance; and the incorporation of positive thinking to the repertoire of cognitive strategies and the decrease of negative thinking. *Third Stage/Long-term Sobriety (4–10 years)*: stabilization of negative emotions; a progressive increase of psychological wellbeing and satisfaction with life, matching control subjects’ scores; and decrease of avoidance strategies use. *Fourth Stage/Very long-term recovery (>10 years)*: predominance of satisfaction with life and psychological wellbeing; autonomy and self-acceptance reach matching values to control subjects; and prevailing of distraction and positive thinking coping strategies. These stages can be comparable to the ones described by the Betty Ford Institute panel (70), which are mainly based on the common experiences of persons in recovery. Considering the diffuse literature consensus on this topic, they carried out a first effort to describe the duration and sobriety stability in the following phases. *Early Sobriety:* a sobriety period of at least 1 month and less than 1 year; *Sustained Sobriety:* that lasts at least 1 year but less than 5 years; and *Stable Sobriety:* over a 5-year period.

**Table 7:** Recovery enhancement in AUD and achieving values similar to a control sample proposed stages of recovery and the corresponding components of wellbeing.

	Early recovery (0–1 years)	Sustained recovery (1–4 years)	Long-term recovery (4–10 years)	Very long-term recovery (>10 years)
Quality of life	Physical quality of life	Psychological quality of life	Social relations quality of life	
Psychological wellbeing	Marked improvement in personal growth	Increase in personal growth and purpose in life	Enhancement of environmental mastery and satisfaction with life	Achieving autonomy and self-acceptance
Negative emotionality	Relevant improvement in anxiety symptoms	Relevant improvement in depression symptoms and experiential avoidance Anxiety improvement stagnates	Negative emotions stabilization	
Coping strategy	Use of distraction and avoidance	Increase in distraction, avoidance, and positive thinking use Decrease in negative thinking	Decrease of avoidance use	Distraction and positive thinking use stabilize

On another note, the significant differences in quality of life and psychological dimensions found between patients and controls can bring more light upon the relation of these variables with abstinence but also their slow progress over time. In other words, people with AUD need long periods of abstinence for their quality of life and wellbeing to change significantly. This might be in line with other findings (16, 25, 61). In the same way, our results are similar to those observed by Kelly et al. (16), obtained from a community sample. Authors indicated that the quality of life continuously improved over the first 11 years after ceasing consumption, and it was similar to the control population after 15 years of abstinence. They first reached similar scores to controls in physical health, followed by psychological health and social relations at 10 years of abstinence. In this way, our results, in a similar manner to other research (71), support the consideration of recovery as a slow process and are in line with the recommendations of the Betty Ford Institute (70) and SHAMSA (7) to include quality of life and wellbeing indicators for recovery.

In regard to psychological wellbeing and its improvement with abstinence, our results concur with other findings (27, 28, 72–75). However, in our study, recovery dimensions seem to be slower, especially for autonomy and self-acceptance, that also match control sample values with recovery periods over 10 years.

With respect to negative emotionality, our findings concur with our previous study. The fact that stabilization of recovery does not happen until patients reach abstinence periods superior to 5 years and those patients do not match control subject scores until 7–10 years of sobriety, along with the relevance of emotional states in relapses (27, 76), makes us think that services should provide for strategies to regulate them at the long-term course of recovery.

Finally, despite the maintenance of abstinence, statistically significant differences have been found between the group with AUD and the control group in impulsivity scores (BIS-11) and positive relationships (PWBS). Impulsivity is a heterogeneous personality and behavioral construct, consistently identified as a trait in substance use disorders, including AUD. Moreover, impulsivity characteristics frequently overlap with various alcohol dependence symptoms, such as unplanned and uncontrolled drinking, despite the negative consequences. The impulsivity role in the initiation and progress of addictive behaviors has been previously highlighted by the literature (77, 78). Our group previously explored the impulsivity role in recovery, and, contrary to other findings showing a decrease in impulsivity after several months of abstinence (28), we found that AUD patients have significantly higher impulsivity scores compared to controls, even after 4 years of abstinence maintenance (28). This might be due to impulsivity characteristics as stable traits (as measured by BIS-11). Despite the possible reductions in impulsive behaviors over time and along the recovery period, trait impulsivity might remain as a personality factor. Additionally, possible inconsistencies across studies regarding impulsivity changes across time could also be related to the clinical characteristics of the samples and the heterogeneous distribution of impulsivity across the population. Regarding the social domain, it is noticeable that AUD patients show similar scores to controls after 4 years of abstinence in social relations measured by the Quality of Life Questionnaire (WHOQOL-BREF), whereas positive relations evaluated by PWBS do not show any relation with abstinence time. Moreover, AUD and control groups maintain statistical differences over time in positive relations. This particular result might indicate difficulties in managing particular characteristics of social relations in AUD patients during the recovery process. One possible explanation for the discrepancy with the WHOQOL-BREF social relationships may be related to its different conceptual features. WHOQOL-BREF provides an overview of general satisfaction with personal relationships, social support, and sexual activity, whereas PWBS’s positive relations attempt to capture specific aspects of social interactions, such as reliance, stability of the social relation, and feeling understood by others. Thus, PWBS’s positive relations might reflect specific characteristics of social interactions where AUD patients might encounter more difficulties and possible challenges. Nonetheless, the particular and detailed aspects of social interactions, benefits, and other characteristics should be further analyzed and differentiated in future studies along several stages of the recovery process.

In summary, the results indicate that the most pronounced changes happen during the first 5 years of abstinence. However, the time needed to reach standardized scores (similar or equal to healthy individuals) in quality of life, wellbeing, and negative emotionality can be fairly more extended since, in our data, individuals with AUD seem to take more than 10 years to match similar values in these dimensions, compared to a control sample. Taking into consideration the relevance of recovery programs based on values [12-steps and Help-yourself, Help-us initiatives (28, 72)] for the self-help group consolidations and their extended time periods, recovery comprehension could benefit from a broadening of the therapeutic stages. At least this could be the case for patients with more severe AUD, beyond the 5-year period of abstinence conceptualized as stable recovery, as proposed by the Betty Ford Institute Panel (70). It should be noted that this stage is not empirically established, and it could derive from the available literature and the common experiences of individuals in recovery.

## 5. Key implications for research, politics, and practice

In consonance with the growing acknowledgment of addiction as a public health matter, a series of key political changes have been made to support the expansion of addiction services. In our opinion, there is a growing need for increasing efforts directed to change the addiction paradigm in the public politics field. The focus should be directed toward a more generalized use of what is known as Recovery Oriented Attention Systems (ROSC), characterized by the use of continuous multi-systemic attention and centered on the person. Hereby, the input of the present study to a multidimensional measure of recovery represents a significant opportunity to eliminate an impediment to progress in this field and could, ultimately, serve as a relevant contribution to guide research, public policies, and future practice. In a similar manner to other disorders, substance consumption and recovery are related to sanitary costs and quality of life. By deepening studies, on recovery, we could obtain a more efficient use of the resources.

Moreover, this study could support therapists and other service providers in the clinical field. The sequence of recovery in different psychological dimensions related to the quality of life provides a model for the orientation of healthcare resources and therapeutic strategies toward recovery times: Initially, it requires a focus on the more medical aspects of recovery (detoxification, physical problems related to alcohol consumption), and it should not ignore that life quality dimensions do not stabilize until several years of abstinence have passed; quality of life needs have to be thoroughly considered during the 1st year of recovery by service providers. These 1st years of recovery have a relevant role in alcohol addiction and in self-help association framework since formal treatments have a shorter duration and these associations can accompany the patients during the whole process, while they are still recovering. Moreover, other considerable dimensions, such as the fight against stigmatization, become relevant, knowing that quality of life improvement in interpersonal relations takes a significantly long time to happen.

## 6. Conclusion

The recovery concept implies improvements in quality of life and wellbeing, which are associated with abstinence maintenance. In this way, recovery is presented as a long and slow process, where the most pronounced changes occur during the first 5 years of abstinence. However, indexes of wellbeing and clinical manifestations (such as anxiety, depression, and experiential avoidance) do not seem to reach values similar to healthy subjects until at least 10 years of abstinence.

Moreover, the results point to a differential progress of the contemplated variables. While the physical quality of life seems to evolve rapidly, reaching similar values to controls after 1 year of abstinence (a stage traditionally named as early sobriety), psychological quality of life perception takes a longer time to improve until equaling control subjects’ values, at the 4th year of abstinence. Furthermore, in negative emotionality, wellbeing and relations require more time, with patients reaching similar values to controls at 10 years of abstinence (after the lifestyle changes possibly involved). Regarding coping strategies, recovery also involves greater use of strategies to impede consumption. It seems like, in the 1st year of abstinence, distraction and avoidance strategies show a fast rise, while positive thinking displays a slower but constant increase, that occur beyond 10 years of abstinence.

## 7. Limitations and future perspectives

Among the limitations of this study, the most prominent one is related to its design since it is a cross-sectional study, and this might limit the causal inferences and increase measure errors. One of the inherent limitations of this design is the lack of temporality regarding the association exposition effect, hampering the possibility to know whether abstinence favors the improvement in different psychological dimensions or whether the enhancements in these dimensions facilitate the maintenance of abstinence. It may involve, as Kelly and Hoeppner (3) indicate, a reciprocal relationship.

Additionally, the sample was obtained by recruiting patients that collaborated voluntarily and was not randomized, which might have a possible bias effect. Another limitation can be sample characteristic variability, and though we paired them in gender, age, and educational level, we could not cover other sociodemographic variables. In addition, the number of control subjects was inferior to patients, which limits the statistical power to detect significant differences between groups.

Lastly, different variables have been studied by intragroup comparisons, which allowed us to know more about dimensions evolutions; however, this strategy might implicate limitations when comparing dimensions and their changes. In this way, it would be of interest to know the relation between the different dimensions along the recovery process. Exploring this interaction might help to understand how they might modulate one another or how the change in one of them can facilitate the change in others. All this could allow for an integral perspective that goes beyond the abstinence relevance, and it would contribute to recommendations for the general practice. It would also be relevant to investigate what other factors are affecting the course and slowing down the process, such as stigmatization.

## Data availability statement

The original contributions presented in the study are included in the article/Supplementary material, further inquiries can be directed to the corresponding author.

## Ethics Statement

The studies involving human participants were reviewed and approved by 12 de Octubre Ethics Committee (19/086). The patients/participants provided their written informed consent to participate in this study.

## Author contributions

GR, LE, RJ-B, and AS contributed to conception, design, and implementation of the study. LE, MB, AZ-B, MC-M, AM-M, DP-S, ER-E, JR-D, and MM contributed to the recruitment of the sample. DL, GR, LE, FA, AS, and RJ-B performed the statistical analysis. GR, LE, AS, and RJ-B wrote sections of the manuscript. All authors contributed to manuscript revision, read, and approved the submitted version.

## Conflict of interest

The authors declare that the research was conducted in the absence of any commercial or financial relationships that could be construed as a potential conflict of interest.

## Publisher’s note

All claims expressed in this article are solely those of the authors and do not necessarily represent those of their affiliated organizations, or those of the publisher, the editors and the reviewers. Any product that may be evaluated in this article, or claim that may be made by its manufacturer, is not guaranteed or endorsed by the publisher.
