# Supplementary material for: How, when, and to what degree do people with alcohol dependence recover their psychological wellbeing and quality of life? The Madrid Recovery Project
Source: Front Psychiatry. 2023 Jun 15;14:1130078. doi: 10.3389/fpsyt.2023.1130078 (PMC10313403; doi:10.3389/fpsyt.2023.1130078)
Supplement: Supplementary file 1 [file Table_1.DOCX]

Table 5.1. Selection of the regression model using AIC for Quality of life and Recovery capital

Note. Year: non-transformed years of abstinence variable; Years^2^: quadratic transformed years of abstinence variable: Year^3^: cubic transformed years of abstinence variable; (*) Model selected.

| **Dependent** | **AIC** | **Intercept** | **Age** | **Gender** | **Year** | **Year^2^** | **Years^3^** |
| --- | --- | --- | --- | --- | --- | --- | --- |
| **WHOQOL-Physical Health** | 4.269.892 | 1.351.149 | 0.01679 | -0.44674 | 0.13449 | -0.00612* | . |
| **WHOQOL-Physical Health** | 4.281.422 | 1.341.981 | 0.01593 | -0.45091 | 0.21413 | -0.01697 | 0.00031823 |
| **WHOQOL-Physical Health** | 4.289.911 | 1.381.834 | 0.02055 | -0.45622 | . | . | . |
| **WHOQOL-Physical Health** | 4.301.768 | 1.372.980 | 0.01751 | -0.43867 | 0.02243 | . | . |
| **WHOQOL-Psychological Health** | 5.364.212 | 1.317.931 | 0.01388 | 0.20063 | 0.30096 | -0.01001* | . |
| **WHOQOL-Psychological Health** | 5.377.196 | 1.308.148 | 0.01296 | 0.19618 | 0.38595 | -0.02159 | 0.00033958 |
| **WHOQOL-Psychological Health** | 5.444.540 | 1.353.653 | 0.01506 | 0.21383 | 0.11762 | . | . |
| **WHOQOL-Psychological Health** | 5.581.694 | 1.400.078 | 0.03100 | 0.12183 | . | . | . |
| **WHOQOL -Social Relations** | 7.552.795 | 1.307.091 | 0.02626 | -0.19004 | 0.21046 | -0.00731* | . |
| **WHOQOL -Social Relations** | 7.561.398 | 1.333.171 | 0.02712 | -0.18040 | 0.07660 | . | . |
| **WHOQOL -Social Relations** | 7.572.778 | 1.307.752 | 0.02632 | -0.18974 | 0.20472 | -0.00653 | -0.00002296 |
| **WHOQOL -Social Relations** | 7.578.065 | 1.363.407 | 0.03751 | -0.24032 | . | . | . |
| **WHOQOL -Environment** | 5.036.448 | 1.538.152 | 0.02802 | 0.06355* | . | . | . |
| **WHOQOL -Environment** | 5.056.329 | 1.539.346 | 0.02843 | 0.06118 | -0.00303 | . | . |
| **WHOQOL -Environment** | 5.074.137 | 1.534.319 | 0.02827 | 0.05933 | 0.02277 | -0.00141 | . |
| **WHOQOL -Environment** | 5.093.914 | 1.535.990 | 0.02842 | 0.06009 | 0.00826 | 0.00056850 | -0.00005798 |
| **VCR-Recovery Capital** | 12.683.400 | 4.119.943 | 0.07896 | 122.272 | 0.69422 | -0.02135* | . |
| **VCR-Recovery Capital** | 12.684.729 | 4.071.557 | 0.07146 | 120.442 | 110.640 | -0.07572 | 0.00159 |
| **VCR-Recovery Capital** | 12.715.423 | 4.196.491 | 0.08308 | 125.222 | 0.29868 | . | . |
| **VCR-Recovery Capital** | 12.825.482 | 4.318.780 | 0.12437 | 0.98749 | . | . | . |

Table 5.2. Selection of the regression model using AIC for Psychological Well-Being and Satisfaction with Life

Note. Year: non-transformed years of abstinence variable; Years^2^: quadratic transformed years of abstinence variable: Year^3^: cubic transformed years of abstinence variable; (*) Model selected.

| **Dependent** | **AIC** | **Intercept** | **Age** | **Gender** | **Year** | **Year^2^** | **Years^3^** |
| --- | --- | --- | --- | --- | --- | --- | --- |
| **PWBS- Autonomy** | 13.140.362 | 3.688.458 | 0.11082 | 0.30056 | 0.16082* | . | . |
| **PWBS- Autonomy** | 13.154.503 | 3.662.356 | 0.10989 | 0.28933 | 0.29456 | -0.00724 | . |
| **PWBS- Autonomy** | 13.157.814 | 3.754.678 | 0.13235 | 0.16431 | . | . | . |
| **PWBS- Autonomy** | 13.174.500 | 3.662.964 | 0.10997 | 0.28955 | 0.28943 | -0.00656 | -0.0000201 |
| **PWBS-Positive Relations** | 14.195.623 | 3.947.165 | 0.13076 | 121.822* | . | . | . |
| **PWBS-Positive Relations** | 14.206.841 | 3.909.867 | 0.11864 | 129.496 | 0.09058 | . | . |
| **PWBS-Positive Relations** | 14.225.277 | 3.894.138 | 0.11808 | 128.819 | 0.17117 | -0.00436 | . |
| **PWBS-Positive Relations** | 14.243.564 | 3.876.909 | 0.11593 | 128.178 | 0.31658 | -0.02369 | 0.0005683 |
| **PWBS-Self-Acceptance** | 14.392.947 | 3.480.683 | 0.14071 | -0.83234 | 0.27184* | . | . |
| **PWBS-Self-Acceptance** | 14.403.759 | 3.440.069 | 0.13894 | -0.84559 | 0.47870 | -0.01116 | . |
| **PWBS-Self-Acceptance** | 14.423.752 | 3.441.248 | 0.13910 | -0.84528 | 0.46883 | -0.00986 | -0.0000383 |
| **PWBS-Self-Acceptance** | 14.443.213 | 3.592.722 | 0.17831 | -107.925 | . | . | . |
| **PWBS-Environmental Mastery** | 14.086.170 | 3.649.622 | 0.14885 | 0.75820 | 0.77409 | -0.02935* | . |
| **PWBS-Environmental Mastery** | 14.098.927 | 3.612.483 | 0.14408 | 0.73938 | 108.939 | -0.07120 | 0.00123 |
| **PWBS-Environmental Mastery** | 14.129.771 | 3.756.560 | 0.15308 | 0.78867 | 0.22763 | . | . |
| **PWBS-Environmental Mastery** | 14.158.337 | 3.850.957 | 0.18480 | 0.55585 | . | . | . |
| **PWBS-Purpose in Life** | 13.734.150 | 3.509.174 | -0.07244 | -0.23121 | 111.506 | -0.09632 | 0.00218* |
| **PWBS-Purpose in Life** | 13.740.023 | 3.574.797 | -0.06373 | -0.19108 | 0.55666 | -0.02217 | . |
| **PWBS-Purpose in Life** | 13.761.454 | 3.655.348 | -0.05999 | -0.15851 | 0.14405 | . | . |
| **PWBS-Purpose in Life** | 13.763.803 | 3.714.671 | -0.03988 | -0.29782 | . | . | . |
| **PWBS-Personal Growing** | 13.699.984 | 3.381.213 | -0.06047 | 163.465 | 134.781 | -0.05199* | . |
| **PWBS-Personal Growing** | 13.707.181 | 3.334.027 | -0.06752 | 161.842 | 174.938 | -0.10513 | 0.00156 |
| **PWBS-Personal Growing** | 13.895.894 | 3.569.926 | -0.04938 | 166.717 | 0.37883 | . | . |
| **PWBS-Personal Growing** | 14.016.237 | 3.725.526 | 0.00497 | 126.693 | . | . | . |
| **SWLS-Satisfaction With Life** | 12.732.353 | 1.917.365 | 0.09116 | 0.58153 | 0.67097 | -0.01784* | . |
| **SWLS-Satisfaction With Life** | 12.749.840 | 1.900.528 | 0.08906 | 0.57526 | 0.81307 | -0.03673 | 0.0005553 |
| **SWLS-Satisfaction With Life** | 12.752.299 | 1.981.669 | 0.09345 | 0.60920 | 0.34150 | . | . |
| **SWLS-Satisfaction With Life** | 12.917.115 | 2.122.283 | 0.13916 | 0.31987 | . | . | . |

Table 5.3. Selection of the regression model using AIC for Negative Emotionality and Impulsivity

Note. Year: non-transformed years of abstinence variable; Years^2^: quadratic transformed years of abstinence variable: Year^3^: cubic transformed years of abstinence variable; (*) Model selected.

| **Dependent** | **AIC** | **Intercept** | **Age** | **Gender** | **Year** | **Year^2^** | **Years^3^** |
| --- | --- | --- | --- | --- | --- | --- | --- |
| **Hamilton Anxiety** | 13.039.595 | 1.104.029 | -0.01229 | 290.540 | -154.155 | 0.12906 | -0.00299* |
| **Hamilton Anxiety** | 13.086.121 | 1.013.290 | -0.02361 | 287.160 | -0.77576 | 0.02726 | . |
| **Hamilton Anxiety** | 13.149.867 | 915.018 | -0.02711 | 282.931 | -0.27225 | . | . |
| **Hamilton Anxiety** | 13.235.846 | 802.919 | -0.06355 | 305.997 | . | . | . |
| **Hamilton Depression** | 14.231.893 | 1.487.338 | -0.11498 | 163.286 | -193.777 | 0.16772* | -0.00400 |
| **Hamilton Depression** | 14.295.978 | 1.366.110 | -0.13011 | 158.770 | -0.91467 | 0.03171 | . |
| **Hamilton Depression** | 14.356.039 | 1.251.812 | -0.13418 | 153.852 | -0.32905 | . | . |
| **Hamilton Depression** | 14.445.452 | 1.116.326 | -0.17822 | 181.730 | . | . | . |
| **BIS-11-Total. Impulsivity** | 18.179.844 | 4.498.632 | -0.34756 | 117.314* | . | . | . |
| **BIS-11-Total. Impulsivity** | 18.185.866 | 4.590.288 | -0.31988 | 0.99749 | -0.22496 | . | . |
| **BIS-11-Total. Impulsivity** | 18.186.418 | 4.697.378 | -0.31365 | 106.951 | -0.79419 | 0.03278 | . |
| **BIS-11-Total. Impulsivity** | 18.200.988 | 4.754.398 | -0.30718 | 108.446 | -128.706 | 0.10108 | -0.00212 |
| **AAQ-II. Experiential avoidance and psychological inflexibility** | 16.247.570 | 3.703.044 | -0.18240 | 283.751 | -285.383 | 0.22287 | -0.00464* |
| **AAQ-II. Experiential avoidance and psychological inflexibility** | 16.291.262 | 3.562.303 | -0.19996 | 278.508 | -166.604 | 0.06497 | . |
| **AAQ-II. Experiential avoidance and psychological inflexibility** | 16.457.827 | 3.328.102 | -0.20831 | 268.430 | -0.46607 | . | . |
| **AAQ-II. Experiential avoidance and psychological inflexibility** | 16.557.643 | 3.136.193 | -0.27068 | 307.917 | . | . | . |

Table 5.4. Selection of the regression model using AIC for Coping Strategies

Note. Year: non-transformed years of abstinence variable; Years^2^: quadratic transformed years of abstinence variable: Year^3^: cubic transformed years of abstinence variable; (*) Model selected.

| **Dependent** | **AIC** | **Intercept** | **Age** | **Gender** | **Year** | **Year^2^** | **Years^3^** |
| --- | --- | --- | --- | --- | --- | --- | --- |
| **CBI-Positive thinking** | 9.651.058 | 2.736.203 | 0.06923 | 119.661 | 0.64517 | -0.05991 | 0.00156* |
| **CBI-Positive thinking** | 9.658.615 | 2.807.178 | 0.07624 | 123.880 | 0.11683 | . | . |
| **CBI-Positive thinking** | 9.668.703 | 2.784.082 | 0.07535 | 122.249 | 0.23736 | -0.00647 | . |
| **CBI-Positive thinking** | 9.681.261 | 2.855.390 | 0.09237 | 115.385 | . | . | . |
| **CBI-Negative thinking** | 10.869.993 | 1.877.469 | -0.00466 | 0.07468 | -0.52216 | 0.01590* | . |
| **CBI-Negative thinking** | 10.876.762 | 1.843.415 | -0.00892 | 0.05154 | -0.23306 | -0.02195 | 0.00110 |
| **CBI-Negative thinking** | 10.891.810 | 1.820.808 | -0.00677 | 0.02327 | -0.22591 | . | . |
| **CBI-Negative thinking** | 10.981.880 | 1.727.954 | -0.03785 | 0.17968 | . | . | . |
| **CBI-Distraction** | 11.760.020 | 1.298.726 | 0.03686 | 163.108 | 158.557 | -0.11934 | 0.00284* |
| **CBI-Distraction** | 11.802.073 | 1.386.205 | 0.04758 | 171.050 | 0.84060 | -0.02196 | . |
| **CBI-Distraction** | 11.838.348 | 1.464.661 | 0.05040 | 179.564 | 0.43116 | . | . |
| **CBI-Distraction** | 12.092.604 | 1.643.734 | 0.11075 | 150.836 | . | . | . |
| **CBI-Avoidance** | 7.133.427 | 780.869 | -0.02521 | 0.02441 | 0.83883 | -0.09313 | 0.00244* |
| **CBI-Avoidance** | 7.312.529 | 856.098 | -0.01565 | 0.07653 | 0.19981 | -0.00941 | . |
| **CBI-Avoidance** | 7.320.335 | 899.831 | -0.01090 | 0.08947 | . | . | . |
| **CBI-Avoidance** | 7.336.374 | 889.683 | -0.01431 | 0.10643 | 0.02461 | . | . |
